# Competition

**Published:** 1881-07

**Authors:** J. W. Lambert

**Affiliations:** Goshen, Indiana


### Domestic Correspondence.

### ARTICLE IX.

### COMPETITION.

MESSRS. EDITORS :—I indite this article with the hope of calling forth a similar comment from others of our profession. The subject in hand is of vital interest to all physicians and surgeons practicing their calling outside of the classic walls of their medical *alma mater.* I do not wish to cast a stone at any particular college or medical uncle who took our cash for wise instructions, dignifiedly imparted, when we of the common herd were in the embryo state. We always will hold them in remembrance dear, and as long as the learned and skilled professor will remain true to his office of imparting medical lore and absorbing legitimate clinical material, then, so far, I say amen. But, for a man occupying any chair at the shrine where all budding M.D.'s for the stipulated time, pay devotion and cash, for him to descend and compete with his own and the fledglings from other institutions, who, after years of patient study and constant practice, have ripened into able surgeons—this fact is what prompts this article. My revered father and my honored preceptors taught me to heed the established code of ethics, and, until I began active practice, I always supposed that the fountain head of professional ethics was at our medical schools ; but when I speak for myself I know personally, that I only echo the same experience for others of the country laity. For example, and to illustrate the workings of competition from high sources backed by the high prestige of a special chair in an established college: I was in the office of Dr. ——, in a neighboring town, where a patient presented himself for examination. Case, necrosis of the os-humeri, requiring surgical interference. The doctor was skilled in that branch of surgery, perfectly competent to operate ; patient well able to pay proper fee. Patient inquired amount of same, and he himself set a day for the operation, but failed to appear. I made it my business to ascertain the reason. Found that he had become alarmed, took flight to the city, had consulted the professor, who concurred in diagnosis and was informed of amount of surgeon's reasonable fee for operation at home. Professor deliberately competed and took in the lucre. I was consulted by a gentleman with a diseased optic, case for immediate enucleation. Advised operation, and stated reasons so that patient could understand. He appreciated the importance of the information and set a near day for the operation, but failed to appear. I bided my time, and discovered that some one had advised him *to go to college where they would take it out for nothing.* This patient was wealthy and my fee was moderate. He acknowledged to me afterward, that he had expected to save fee by free operation at the college clinic, but decided not to make a show of himself, and had determined to return to me. When the chair of ophthalmology learned this fact, it bent down to inquire the amount of my fee, deliberately competed, and the lucre was kept out of home circulation.

I could enumerate over twenty cases of recent and more active competition than the above, for I spared no time or expense in order to get at the facts and satisfy myself truly, why consulting cases seldom return from their trip unplucked after crossing the spider's portals. In this manner does the country doctor's *honorarium* become a *non est.* During my last visit to the city one source of amusement was to notice the workings of the professional man from high to low stations, and I find the greed for coin overtops all points that can be strained. I hope all of our able country physicians and surgeons will awake to these facts and consult their own interests by more unanimity of feeling and recognition of each other's worth, and assist each other to combat this tendency to absorb our legitimate cases, especially those inclined to flee to the cavernous maw of the delusive college clinic. Any physician who has had experience at a college hospital where he could get an idea of each professor's private income from pay cases, is aware that a major portion of such income is derived from an unfailing supply of patients from the country. Now too often is it a fact that cases are referred to professor so and so, in preference to turning the same over to our competitor at home who is perfectly competent to properly attend the same, and who would give closer and more undivided care to the case than the patient could possibly obtain at the professor's crowded clinic. Surely the medical millennium would be at hand if this *odium medicum* could be overcome. At the founding of a medical college one principal card to induce students to attend is the charitable free clinics, and a grand good school it is. The clinic is advertised to be supported by the State and materialized by the abundant paupers. This is the correct and proper source to draw from, for it does not interfere with the income of the special physician or surgeon either of the city or country. But when the free clinic is open to the whole and adjoining States regardless of the ability to pay, of many applicants, then do we gnash our teeth over the loss of that case and the needed fee; also, not to speak of the absorption of the pluckings by the professor at his elegant private office where the sufferer is impressed with the belief that therein is condensed the quintessence and *ultima thule* of medical skill.

Do you not see my dear country doctors wherein lie our grievances? We set our professional hook baited with half a life's hard study; little cases come and nibble, then a big bite. We have the prize half landed (in our mind), when lo, the shark of the college clinic gobbles it up! Let us reform and protect ourselves. If Dr. A. does not feel at home with a case, send it to Dr. B. across the street, who is just the man for the particular case. Do this in order to uphold the dignity and honor of our home and country practice. Please do not consign a patient to the cramped wards of a hospital when they can secure as good services at home, with the benefit of country air and wholesome diet. Every community is blest with a competent physician or surgeon, whose door cheerfully opens at the knock of even the suffering poor. But I had better close this communication for I may have offended some one; if so, I beg pardon, for I have only jotted down a few of my observations on this internal competition and jealousy among our profession, and think it time reform should set in. This article may not meet the approval of every reader, but, *damnant quod non intelligunt*.

Goshen, Indiana. DR. J. W. LAMBERT.

